# Impact on the German asymptomatic screening strategy based on actual user data from SARS-CoV-2 test centers

**DOI:** 10.1038/s41598-023-47262-x

**Published:** 2023-11-15

**Authors:** Marcus Grohmann, Janina Grosch, Beate Conrady, Lena Schomakers, Anna Kristina Witte

**Affiliations:** 1 HTK Hygiene Technologie Kompetenzzentrum GmbH, Buger Str. 80, 96049 Bamberg, Germany; 2 Department of Veterinary and Animal Sciences, Faculty of Health and Medical Sciences, University of Copenhagen, 1870 Frederiksberg, Denmark; 3 Complexity Science Hub Vienna, 1080 Vienna, Austria

**Keywords:** Public health, Health policy

## Abstract

Since March 2021, Germany has been providing cost-free severe acute respiratory syndrome coronavirus 2 (SARS-CoV-2) antigen tests, and many day-to-day activities following the lockdown have required negative test results. Yet it remains unclear how tests have been used and whether there are patterns connected to mitigation measures. We analyzed over 50,000 anonymized records from eight test centers in a typical medium-sized city, with one of them remaining open continuously from March until December 2021. The centers exhibit distinct patterns of visitor types, with the majority tested only once in the investigated period. Individuals who underwent repeated testing tended to favor the same location. A preference for spontaneous testing grew in proportion to the availability of spare tests. Visitors aged 18 to 30 years were distinctly overrepresented compared to the local demographic. A negative binominal model showed that implemented mitigation measures had an impact on the number of tests conducted. Cost-free testing in private facilities was implemented into the German complementary screening strategy, aiming to achieve weekly population-wide testing. This study demonstrates these facilities were rarely used for regular testing but rather for meeting requirements of certified tests. The results should aid authorities in making future decisions regarding infection control.

## Introduction

As measures against the spread of the severe acute respiratory syndrome coronavirus 2 (SARS-CoV-2), non-pharmaceutical interventions in lockdowns, such as mask wearing, business closures, or contact reduction have been shown to be effective^1–3^. A further measure in controlling the pandemic of the coronavirus disease 2019 (COVID-19) is vaccination which was implemented late 2020^4^. Testing for SARS-CoV-2 infections followed by the isolation of infected individuals is another approach as often a few people are responsible for a high number of SARS-CoV-2 transmissions^5^. All of these measures have been combined into a multilayer approach^6^.

As reference diagnostic assays for SARS-CoV-2 detection, the World Health Organization has implemented methods based on the amplification of nucleic acids^7^. The demand for Point-of-Care (PoC) testing to detect SARS-CoV-2 independent of laboratory environments has led to the further development of nucleic acid testing, immunoassays, and biosensor testing. The less sensitive but faster and more affordable antigen detection methods were the first authorized rapid tests and can complement testing^8^. However, when using this method for broad application testing, the limited sensitivity may result in undetected infected individuals. The false-positive tests result in unnecessary isolation, as well as high costs in extensive test strategies^9^.

In March 2021, Germany extended its multilayered approach of public health measures to control the pandemic by implementing a complementary, voluntary screening strategy that would continue until the end of June 2022^10^. It aimed for the early identification of infections in asymptomatic individuals by providing easily accessible, free of charge antigen testing. These tests were made available to all German residents on a voluntary basis at least once per week. Models of large-scale screening have shown effectiveness when there is high compliance in the population for frequent testing^11–14^. This weekly testing strategy aligns with recommendations made by Peto et al.^15–17^.

To achieve a high level of testing compliance, the German government introduced various measures depending on local incidence rates. By the end of 2020, local vaccination centers have been established and offering cost-free vaccinations in accordance with the national immunization strategy^18^ On the state level, Bavaria declared the so-called *Katastrophenfall* (disaster situation)^19^ in the winter of 2020/2021, leading to the closure of restaurants and recreational and cultural facilities due to high infection rates. In spring 2021, these establishments were gradually reopened (i. e. restaurants reopened starting on10/05/2021) as infection rates declined. The requirements for visiting the aforementioned facilities changed multiple times during 2021. In spring, a negative test result was required for everyone (17/04/2021–27/05/2021). On June 6, 2021, the disaster situation came to an end and throughout the summer months no negative test result was needed to access to restaurants or engage in other activities. In late summer, a negative test result was only required for indoor activities for individuals without vaccination or those not yet recovered from COVID-19 (23/08/2021–10/10/2021). With decreasing infection rates and increasing vaccination coverage, the testing strategy changed to a paid service (11/10/2021–12/11/2021), whereas vaccination against SARS-CoV-2 remained free of charge. Starting on 18/10/2021, a negative test result was required for all cultural activities and restaurants for individuals who were neither vaccinated nor had recovered. Already by the end of November an increasing number of infections even among fully vaccinated people, prompted a return to the policy of providing free testing. Due to the increasing number of infection rates in November 2021, the disaster situation was declared again (10/11/2021) and a new workplace regulation was implemented (24/11/2021): people without vaccination or recovery required to present a negative test result at their workplace. The free-of-costs testing strategy was maintained until July 2022 despite higher vaccination rates and the emerge of new virus variants. Since then, free testing has only been available for patients, visitors, and healthcare employees as well as for people with a recent SARS-CoV-2 infection to facilitate the end of their isolation^20^.

We demonstrated in a previous study^21^ that different test centers were used by different people. This study included a, 12 week analysis from eight test centers for SARS-CoV-2 antigen testing in Bamberg, a typical medium-sized city^22^ in Bavaria^21^. This independent city with a population of 77,749 at the end of 2021 has a catchment area of more than 350,000 residents with an average age of 43 years. The unemployment rate at 4% at the end of 2021, is slightly lower than the national (5.1% at the end of 2021) and the European average (6.4% at the end of 2021). In the present study, we analyzed a considerably longer period of 8 months. Our objectives were as follows: (1) to determine the frequency of testing among individuals, (2) to assess whether they visited one or multiple testing locations, (3) to identify the most common transitions between locations, and (4) to confirm differences between test centers. Additionally, we sought (5) to detect temporal changes, particularly during the three weeks of fee-based testing, and (6) to identify factors influencing the number of performed SARS-CoV-2 tests. Finally, (7) we aimed to assess the extent to which the prediction of the number of tests performed, corresponds to the actual observed number of tests by using all statically significant variables and individual variables. This study inspects the usage of a large-scale screening program addressing asymptomatic individuals over a prolonged period associated with a change of various conditions. It aims to assist authorities with future decisions.

## Results

### Visitors in eight different test centers—differences, returners, interchanges

We evaluated the usage of PoC antigen testing capacity provided to the public at eight locations in a medium-sized European city (Bamberg, Germany) in accordance with national COVID-19 regulations, starting in April 2021. In total, 51,917 tests administered to 32,879 visitors, have been recorded from 29th March to 30th November 2021. The first PoC tests were offered at the location near the central bus station. This service was supplemented by providing tests at several locations in the city using a mobile test center in the form of a public bus from 15th April to 19th June. To ensure easy access and meet expected demand, additional six test centers were established. These centers were strategically located along the main traffic routes in each city sector and had varying opening hours. Test centers gradually closed as demand decreased during the summer months, beginning in June (Supplemental Fig. S1, a map for the test centers has been published previously^21^).

In summary, we found that visitors differed among the individual test centers in terms of age, origin, the proportion of spontaneous tests, and tests per hour (Supplemental Fig. S2).

**Figure 1:**
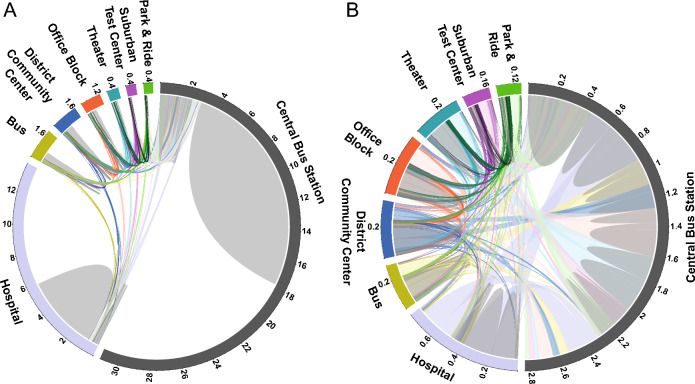
Chord diagram of all tests and tests exclusively from visitors changing location. (**A**) Tests are arranged around the circle and connected using links with respective colors to tests from the same visitor. Each combination of locations is represented by a different color (e.g. Bus and Central Bus Station in yellow). Links showing visitors returning to the same locations are drawn in grey. Tests from one-time visitors are shown without any link. (**B**) Same visualization as a without the tests of one-time visitors and visitors that exclusively returned to the same location. Numbers of tests are indicated in 1000 increments clockwise around the outside of the circle.

Depending on the location, test capacity and operating hours, the number of tests for each test center varied widely (Fig. 1A). The majority of tests were performed at the Central Bus Station (31,169), followed by the Hospital (13,891). Distinctly fewer tests were conducted at test centers with shorter opening hours: Bus (1906), District Community Center (1685), Office Block (1295), Theater (733), Suburban Test Center (636) and Park & Ride (602).

Most visitors (76%) used any of these eight test centers only once within the investigated period. Only five individuals underwent testing on a weekly basis. The lowest proportion of one-time visitors, accounting for 39% of all tests, was at the Theater, while the highest proportion, at 66%, was at the Park & Ride (Fig. 1A, tests without any connections within the circle).

Another majority of tests were used by returners who repeatably used one specific location, as visualized by the grey connections (Fig. 1A flanking the one-time visitors). The lowest proportions of tests by returners were observed in the centers with the lowest number of visitors: the Park & Ride and Suburban Test Center, with 7% and 8% respectively. Tests by returners accounted for 48% at the Central Bus Station and thus, making it the test center with the highest proportion of tests used by returners.

A closer examination of tests performed by individuals who used multiple test centers is provided in Fig. 1b: Visitors who utilized several test centers often did so repeatedly (grey connections within the own test center). The use of only two test centers was more common than more frequent interchanges. The most frequent combinations of different test centers, represented by thicker connections, were the Central Bus Station each with the Hospital, with the Office Block and with the Theater, despite the Theater’s low number of total tests. Interchanges between the less frequently visited test centers were rare. Test centers with the highest number of tests and longest operating periods (Central Bus Station and Hospital) had the lowest proportion of interchanges, accounting for 9% and 6% of the tests, respectively. Among the smaller test centers, it is noteworthy that the Bus had a relatively low percentage of interchanging tests (15%) compared to the other small test centers. The absolute number of interchanging tests was even lower than that of the District Community Center (22%), Office Block (31%), and Theater (43%).

### Temporal progression of antigen test usage with changing conditions

**Figure 2:**
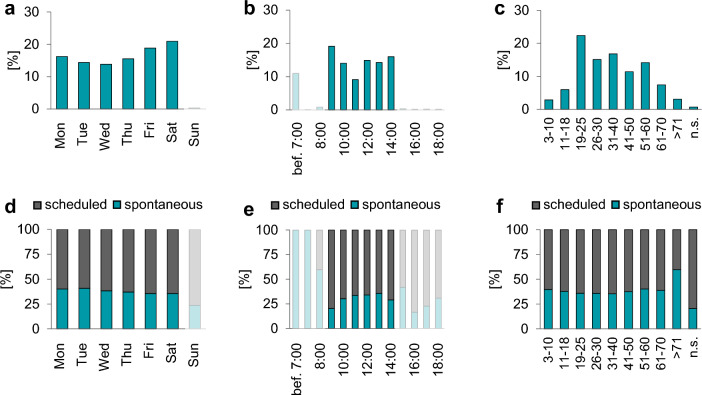
Utilization of the test center ‘Central Bus Station’. (**a**) Percentage of tests on weekdays. (**b**) Percentage of tests during opening hours (the tests before 7.00 are the retrospectively manually recorded tests). (**c**). Percentage of tests across the visitors’ age groups. (**d**–**f**). Ratios of spontaneous (turquoise) and scheduled (grey) appointments depending on weekday, testing hours and ages. n.s.: not specified. Transparent highlighted are times that were typically not used for testing, but there are data available (due to exceptions, or, in case of data before 7 am, retrospectively recorded tests).

One primary objective of this analysis was to evaluate the temporary progression of free PoC testing under changing conditions during the pandemic. The test location at the Central Bus Station remained in continuous operation throughout the observation period and was thus subjected to more detailed analysis. The majority of tests were performed on Fridays (19%) and Saturdays (21%) (Fig. 2a) with peak testing at the start (19%) and before closing (16%) the center (Fig. 2b).

**Figure 3:**
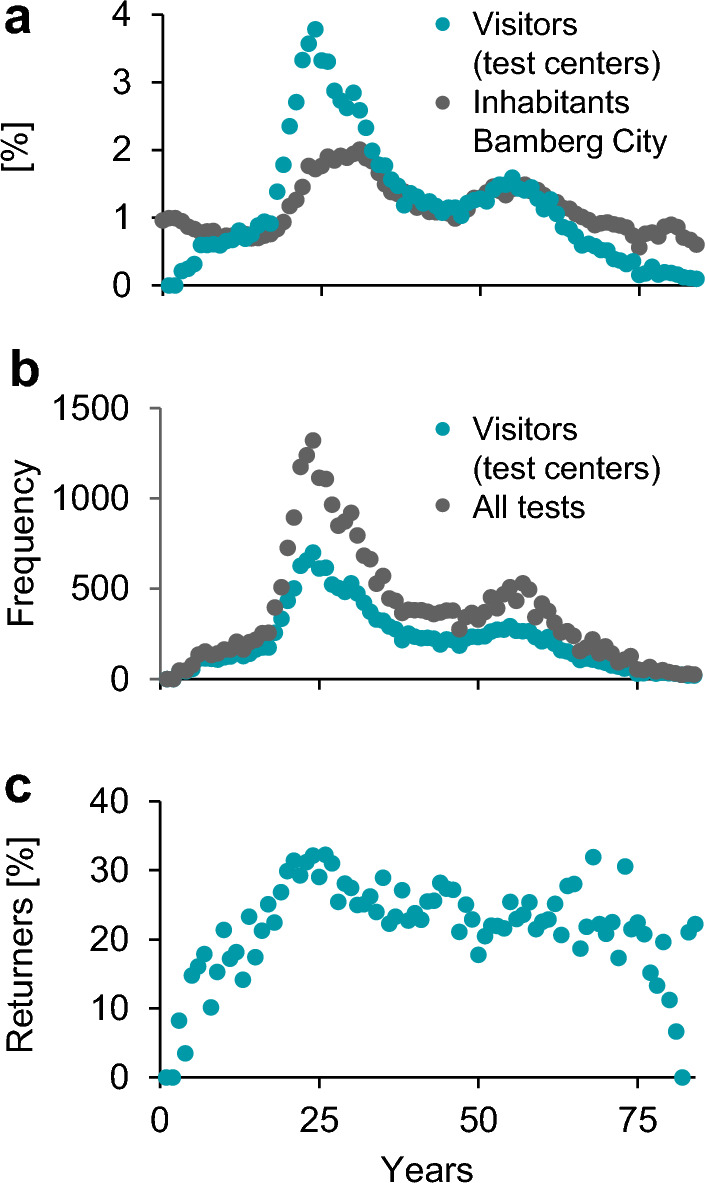
Age specific data for the Central Bus Station test center. (**a**) Age distribution of the demographic from the city Bamberg (grey) and the Central Bus Station’s visitors (turquoise). (**b**) Frequency of all visitors (turquoise, each visitor is counted once) and all tests (grey, visitors were counted as often as they were tested). (**c**) Proportions of returners for each age.

The largest group of visitors consisted of young adults aged 18 to 25 (Fig. 2c). When comparing these proportions to the registered residents of the city, the data indicates that young adults used the tests distinctly more often than other age groups. Conversely older people (~ aged 60 and older) used them proportionally less (Fig. 3a). Additionally, the peak in the number of tests is even higher among the younger group than the peak of visitors (Fig. 3b), highlighting the high number of returners in this age group (Fig. 3c). The lowest proportions of returners were among minors (18 years and younger) and elderly visitors (over 80 years).

The majority of tests (62%) were pre-scheduled, without regard to the weekday (Fig. 2d). The exception was visitors older than 71 years, who used the facility more spontaneously (60%) (Fig. 2f). Among the pre-scheduled tests, the highest proportion (80%) occurred at the beginning of the opening hours with the second highest (58%) occurring just before closing (Fig. 2e).

**Figure 4:**
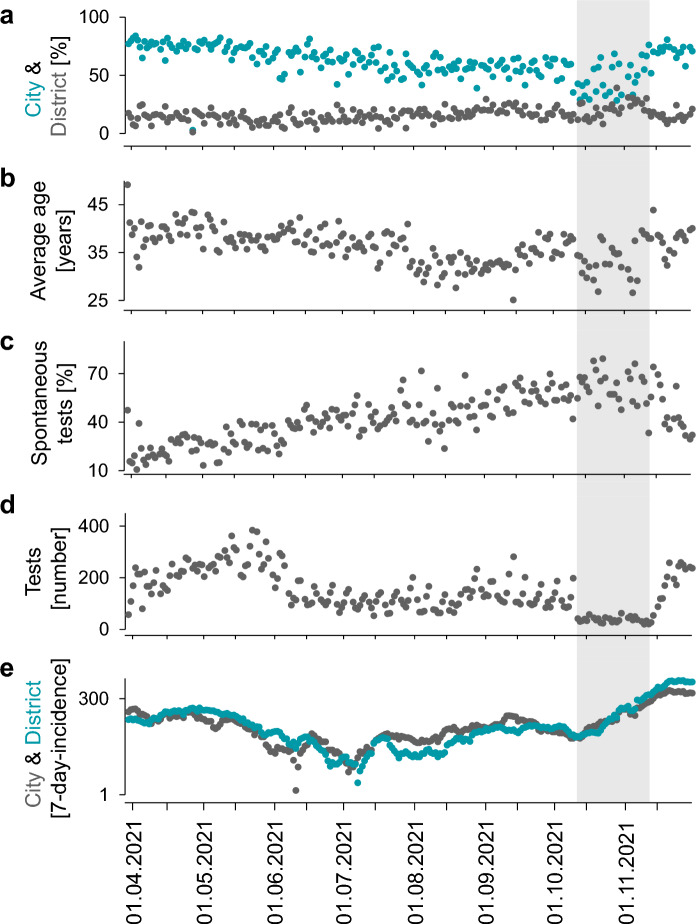
Central bus station—period from end of March until end of November on daily basis. (**a**). Proportion of visitors living in the city Bamberg (turquoise) and its rural district (grey). (**b**) Average visitors’ ages. (**c**) Proportion of spontaneous performed tests. (**d**). Overall number of tests. (**e**). Infection rates of the city Bamberg (grey) and its rural district (turquoise) indicated as 7-day incidence per 100,000 inhabitants (in log scale). The grey area reflects the time where tests were fee-based for most people.

The facility used for this analysis was located in the city center. Throughout the entire period, there was a higher proportion of visitors from the city compared to the urban district which a slight decreased during the summer months (Fig. 4a). The proportion of returners, especially those visited more frequently, was highest among city residents and very low among those from outside of Bavaria (Supplemental Fig. S3). The average age of visitors was lower during the summer school holidays (30/08/2021 until 13/09/2021) (Fig. 4b). The percentage of spontaneous testing without scheduled appointments increased (Fig. 4c) during the period when the number of performed tests and the 7-day incidence rate were lower (Fig. 4d, e). As the 7-day incidence rate increased again in the autumn, the capacity for free tests decreased. General cost-free testing was suspended in late October 2021. The number of tests performed remained relatively low until the costs were covered by the government. This increase in test numbers coincided with a lower percentage of spontaneous testing. (Fig. 4c, d).

**Figure 5:**
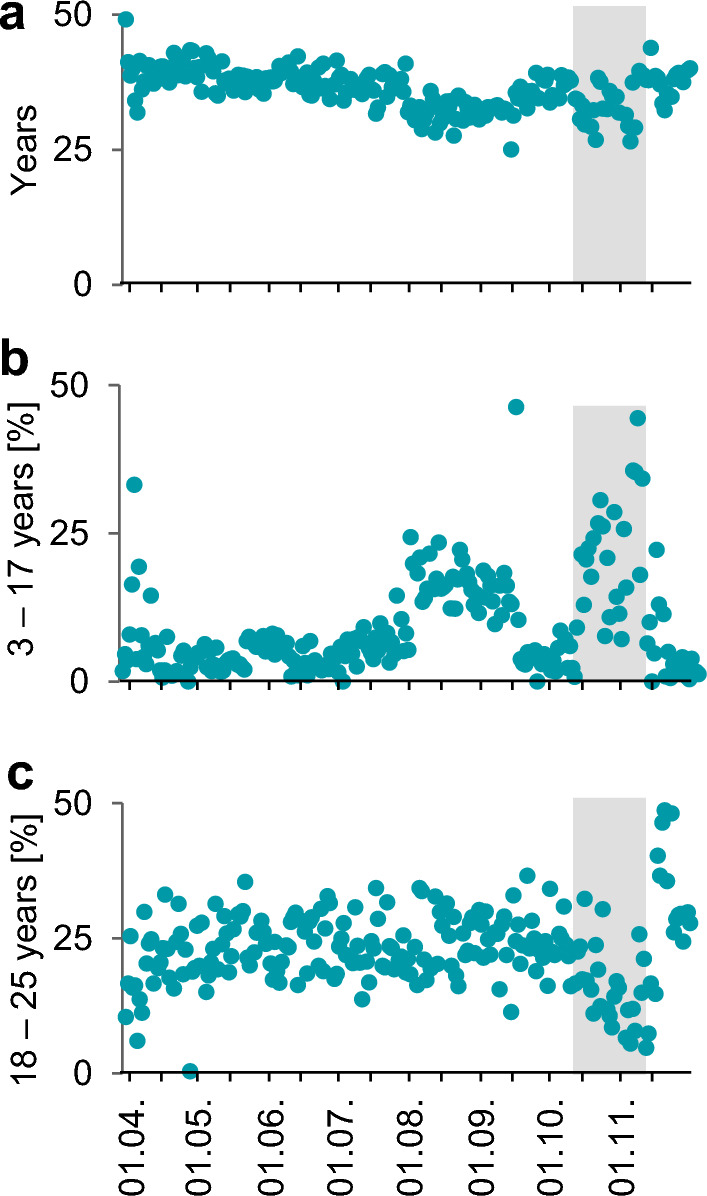
Temporal progression of age-related data in the Central Bus Station test center. (**a**) Average ages of visitors per day. (**b**) and (**c**) Proportion of tests from the 3–17-and 18–25-years old visitors. The grey area reflects the time where tests were fee-based for most individuals.

The younger age of individuals during the summer school holidays (Fig. 5a) noted above clearly coincided with a higher percentage of tests from visitors aged 3 to 17 years (Fig. 5b). However, there was no change in the proportion of young adults (Fig. 5c). The second peak among 3- to 17-year-old visitors occurred during the period when tests were fee-based for individuals aged 18 or older (Fig. 5a, b). Simultaneously, the proportion of visitors aged 18 to 25 dropped considerably (Fig. 5c). In contrast to these two age groups, the proportions of the remaining age groups remained relatively constant (Supplemental Fig. S4).

**Figure 6:**
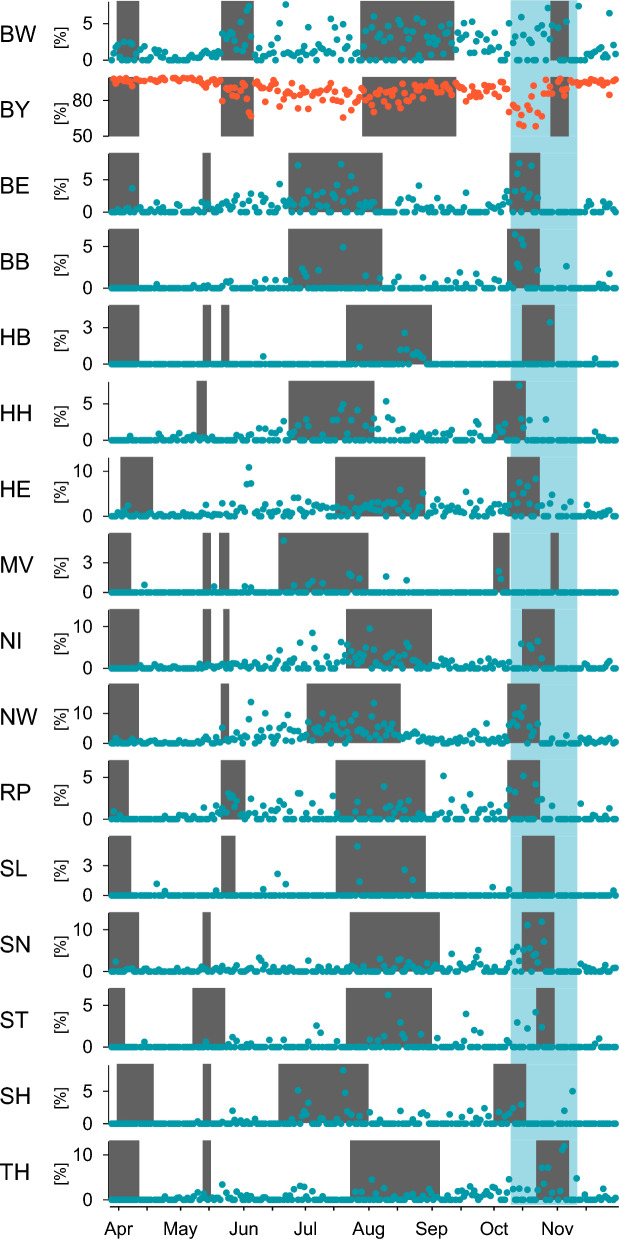
Central bus station—distribution of federal states and their holidays. Visitors were each categorized to one of the 16 federal states according to their postal codes. Holidays in each respective state are shown in dark grey, and the light blue area reflects the time where tests were fee-based for most people. BW: Baden Wurttemberg, BY: Bavaria, BE: Berlin, BB: Brandenburg, HB: Bremen, HH: Hamburg, HE: Hessen, MV: Mecklenburg-West Pomerania, NI: Lower Saxony, NW: North Rhine Westphalia, RP: Rhineland Palatinate, SL: Saarland, SN: Saxony, ST: Saxony Anhalt, SH: Schleswig–Holstein, TH: Thuringia.

Naturally, most of the tests were used by visitors from Bavaria (Fig. 6). With the beginning of the Pentecost holidays (from 25/05/2021 to 04/06/2021), the proportion of visitors from Bavaria decreased, while it increased from other federal states, especially from Baden Wurttemberg, a neighboring state that also had holidays. From that date onwards, tourist accommodation was allowed, and cultural and recreational activities such as theater, cinema, or public pools in Bamberg required negative test results. The proportion of tested individuals from Bavaria remained dominant during the entire investigation period. The proportions of visitors from other federal states increased mainly during the holidays of their respective regions.

### Impact of mitigation measures and weekdays for the prediction of SARS-CoV-2 tests

**Figure 7:**
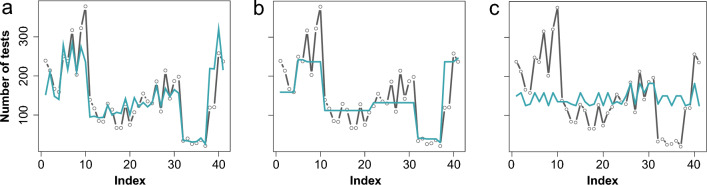
Number of predicted SARS-CoV-2 tests by using the four explanatory variables in the model (**a**), just the implemented mitigation measures in the model (**b**) and the weekday in the model (**c**; turquoise line—model prediction by using training dataset; gray line—test dataset).

The backwards-fitted model includes four explanatory variables that influence the number of SARS-CoV-2 tests at the Central Bus Station test center: The two significant variables are weekdays (*p* < 0.001) and different implemented combined mitigation measures (*p* < 0.001) (D for disaster situation in Bavaria, P for paid testing, W for individuals must be vaccinated, recovered, or negatively tested at the workplace, T for tests required for many activities, and G for gastronomy open). The average age of tested individuals (*p* = 0.07) and the proportion of returners in the test center (more than 15 times; *p* = 0.25) are non-significant predictors (Supplemental Table S5). A strong correlation was identified between mitigation measures and the 7-day incidence (variance inflation factor > 10) and thus, the 7-day incidence was removed from the final model. All measures have a significant positive impact on the number of tests except the measures that included ‘paid testing’. The odds for SARS-CoV-2 tests were 6.71 times higher with the implementation of the measures DWTG and DT and 1.47 or 1.33 times higher on Saturdays or Fridays (Supplemental Table S6). The prediction of the number of tests through the four final variables included in the model is visualized in Fig. 7a. Figure 7b and c indicated a better prediction of the number of performed test via mitigation measures compared to the weekdays.

## Discussion

Our results provide insights into the de facto usage of cost-free large-scale universal SARS-CoV-2 testing. This screening strategy was introduced in Germany in March 2021, during a period of changing COVID-19 incidence and public health policies. To achieve the goal of at least one weekly test per individual, options for screening using antigen rapid tests were made available in the public sector, including schools and educational facilities, in public and private workplaces, in retail sale for home testing and in private testing facilities. In Germany, private providers are only allowed to operate publicly funded test centers after receiving authorization from the public health department. Our dataset represents the bulk of those regional test centers, neglecting other testing categories. These test centers provided certified test results, and, except for a brief period in Nov 21, offered a cost-free service.

During the first 12-week period, no regular testing frequency was reached, as less than 4% of individuals tested more than five times^21^. The observed differences in age composition and the origin of individuals, as well as their booking behavior between test locations, were confirmed throughout the rest of the year. In the initial 12 weeks, the visitors at the hospital were considerably older than in most other test centers. However, during the long-term period, an alignment of the age profile, likely due to changing regulations for hospital visitors during the summer. The trend from the first investigation period was confirmed, with only a small proportion, below 0.5% (110 individuals), visiting 15 times or more in the 8-months period. 76% of the visitors came only once, taking into consideration the limitation of an inflated number of individuals due to typing errors. The higher proportion of individuals testing regularly in the beginning might be attributed to the more frequent necessity of certified negative tests for activities after the lockdown, general higher motivation initially, or a shift to other options such as testing at home or in the workplace. Throughout the 8 months study duration, we found only five individuals out of more than 30,000 who reached the minimum weekly testing frequency. This clearly indicates that private test centers are not favored in achieving a sufficient testing frequency. A model calculating the effectiveness of tests for managing the pandemic in Germany assumed that 40% of tests are conducted in the workplace^23^, supporting this presumption.

Analysis over 8 months demonstrates the dynamics of the test behavior over time. It changed in terms of age composition and the origin of individuals during holidays. Spontaneous testing was preferred when more test capacities were available. This preference could be due to a general inclination for spontaneous testing or a decrease in demand corresponding to a lower 7-day incidence during the summer period. A distinct reduction in tests performed was observed during the fee-based period. Simultaneously, there was a sharp increase in the proportion of minors, as they could still access the tests free of charge.

With respect to weekdays, Saturday has a major positive impact on test frequency in the model approach. More people have free time for activities requiring a negative test result, as well as for testing itself, on Saturdays, since the majority are not working. This is also evident in the high volume of tests performed at the beginning of the opening hours, which aligns with the start of a regular working day. Therefore, offering tests outside regular working hours might increase the utilization of the service. Assessing the influence of the mitigation measures is complex, as they were often implemented simultaneously^2^. Mitigation measures and the correlating 7-day incidence influenced testing behavior, as some activities required a negative test result and became stricter with increasing 7-day incidence, reflecting the rising infection risk. In every combination with other measures, switching to a fee-based operation resulted in a lower volume of tests. All combinations that led to increased requirement for negative test results demonstrated a high number of tests, except for any combination with the fee-based variable. This indicates that the usage of private test facilities is dependent on the requirements for certified tests if they are cost-free. It highlights the necessity of a clear rational for participation in a screening program. This finding contrasts with a survey we conducted within the framework of the test center, where most participants indicated meeting with friends and family as a primary reason for testing^24^. In contrast, a survey from England reported that most respondents primarily underwent work-related testing^25^, but this was in the context of the announcement of a national lockdown. This study was the only other study we found that analyzed data from symptom-free antigen testing. However, they did not go into detail comparing different testing sites, had a shorter study period of 2 months and did not consider temporal progression. Therefore, further work is necessary to gain insights into test usage and to consider the limitations of surveys as a basis for assumptions.

Evaluating the measures implemented to control the pandemic is challenging due to strong temporal overlap and complex interactions. Additionally, the collection, accessibility, and structure of datasets are partly inconsistent between countries, states, and municipalities. Publicly available data about comparable antigen test programs is especially limited. Data about performed tests and positive results were collected by local health departments but are not publicly available. Furthermore, systematic data about test usage at work, school, or in retail sale were missing. Thus, we are confined to our regional dataset, and the comparability to data from other regions is restricted. Moreover, it is unknown how many confirmed SARS-CoV-2 positive cases were detected by cost-free antigen tests. Having an incomplete dataset hampered the evaluation of the measures in Germany, suggesting the need for an improvement in data acquisition in future^26^.

Finally, it should be noted that the diagnostic accuracy of a rapid antigen test depends on various factors, including sampling performance, the viral load of the person being tested, or potential mutations of the virus. However, the focus of our study was on the testing behavior of the population, regardless of the test result. Further work is needed to analyze the effect of false test results (false positive and false negative) on the population.

Overall, detailed insights into the usage of private test centers reveal several key findings: (1) Weekly testing is a rare phenomenon, (2, 3) most individuals who get tested repeatedly prefer the same location, and (4) each test facility exhibits specific characteristics. The duration of the study period allowed for (5) identifying temporal changes and (6) assessing the impact of these changes on the usage of antigen tests. Last (7), the mitigation measures compared to the weekdays can better explain the number of tests performed. These results underscore the importance of systematic data analysis to enable guidance for making appropriate policy adjustments*.*

## Methods

### Test centers

In Bamberg city and its rural district, the local institute HTK offered eight facilities for cost-free SARS-CoV-2 antigen testing^21^. An overview of the open periods of each center is illustrated in Supplemental Fig. S1. The analysis was carried out over the complete operating period, with the longest being at the Central Bus Station from 29/03/2022 until 30/11/2022.

### Data

The data collection procedure for this study was described in our previous work^21^. In brief, individuals could either book a time slot for testing online or visit the test centers spontaneously. They then register either via QR code or through a center employee. All individuals tested at the test centers agreed to a statistical analysis of their data by providing consent through a privacy statement. However, none of those personal data collected during this process was available for the study. Instead, data were provided only in anonymized form, which was created through the message-digest algorithm 5 (MD5) hash function^27^ by the software provider (KALA YOUR LIFE, Bamberg, Germany). Datasets of people who were registered with the online booking system and tested on 26th April were deleted by the test center due to incomplete data privacy statements on the first day of the new registration system. Only the time and test-number were recorded. Datasets with missing data were handled as “not specified”. Datasets without assignable postal codes or with ages less than 3 years or more than 99 years were excluded.

### Analysis

Age: Average and median ages of visitors were calculated for each location. On a daily basis, the average of each test was used.

Region: Visitors were classified into one of the 16 German federal states using postal codes, and, if applicable, into the urban district (‘city’, *Stadt Bamberg*) and the rural district (‘district’, *Landkreis Bamberg*).

Scheduled versus Spontaneous tests: All tests conducted within a timeframe of two hours or less between registration and testing, including all manually registered tests, were considered as spontaneous.

Returner: Individuals that came for more than one test.

Movement between locations: To illustrate the movement of visitors between the locations, we used the R/Shiny application for creating Circos plots^28^. Data of all tests were assigned to each location. Within each locations’ dataset, data were arranged as follows: (I) tests of one-time visitors, (II) tests of returning people who were exclusively tested only at this location and (III) tests of visitors who were tested in several locations. The last group, III, was further categorized into (i) tests of visitors who were tested additionally in other locations only and (ii) tests of visitors who were tested again at the same location and at other locations(s). Lastly, both groups (i and ii) were further arranged into (a) tests of visitors tested in two locations or (b) more than two locations.

### Metadata

The Seven-day incidence (SARS-CoV-2 infection rate per 100,000 citizens) of the city and rural district of Bamberg was obtained from the database of the federal institute *Robert Koch-Institut* (Berlin). The demographic structure of Bamberg was obtained from the *Landesamt für Statistik* (State Office for Statistics). Data about mitigation measures were collected from press releases by the city and district of Bamberg, as well as the webpage of the Bavarian broadcast^29^.

### Statistical analysis

To assess the factors influencing the number of SARS-CoV-2 tests, a negative binomial regression model was used as the variance was larger than the mean. The number of SARS-CoV-2 tests conducted was considered as dependent variable, while the independent variables included 7-day incidence (Bamberg city), mitigation measures (see below), weekdays, average age of tested visitors, and the proportion of returners (> 15 times). A stepwise, backwards factor-selection approach by using log-likelihood ratio tests was applied to compare models with different factor combinations (with a threshold of 0.05) and to include the most relevant variables in the model^30^. The data covered an observation period of 35 weeks and were recorded at the daily level. Additionally, the collinearity of the independent variables of the final model was analyzed using the variance inflation factor. To evaluate the goodness of fit of the final model, the Cragg-Uhler (Nagelkerke) pseudo-coefficient of determination of R-squared (R2) was calculated.

Furthermore, the final model was verified by partitioning the data into the training (70% of the dataset) and the testing dataset (30% of the dataset). We have visualized how good the prediction of the number of SARS-CoV-2 tests are by using all and individual variables (30). All analyses were conducted with the open-source statistical computing environment R version 4.0.5 (R Development Core Team 2021) and the significance level for the statistical analysis was set to *p* < 0.05.

For the investigation period (29/03/2021–30/11/2021), we selected key mitigation measures that were in effect in the city of Bamberg. Unfortunately, most of the mitigation measures were implemented in combination:

1. D: *Katastrophenfall in Bayern*, disaster situation in Bavaria is determined by the Bavarian government between 29/03/2021 and 06/06/2021 and 10/11/2021–30/11/2021
2. P: Fee-based/paid testing period: SARS-CoV-2 PoC Antigen tests must be paid by most people (exceptions: people younger than 18, pregnant & breastfeeding women, people that cannot be vaccinated against SARS-CoV-2 due to medical reasons, SARS-CoV-2 infected people, which need testing for ending isolation, people participating on clinical studies for vaccinations against SARS-CoV-2) 11/10/2021–12/11/2021
3. W: ‘3G am Arbeitsplatz’: People must be (1) vaccinated, (2) recovered or (3) negatively tested at the workplace; controls by employers (24/11/2021–30/11/2021)
4. T: Tests required for many activities (changes between everyone must be tested, only neither vaccinated nor recovered must be tested, only tests for inside activities, only test for not vaccinated/recovered people necessary etc.): 17/04/2021–27/05/2021, 23/08/2021–10/10/2021 and 18/10/2021–30/11/2021
5. G: Gastronomy open: 10/05/2021–30/11/2021

### Ethics declaration

Ethical approval was not required for this study on basis on the following considerations: No personal data were collected to conduct the study. This study includes information freely available in the public domain (infection rates, demographics, public holidays, mitigation measures) on the one hand. On the other hand, the analysis of the datasets from the test centers origin from data that were properly anonymized by the software provider of the test centers and informed content was obtained at the time of original data collection. Furthermore, data collection followed the DFG guidelines on the handling of research data. Due to the anonymous dataset, the study has been granted exemption from ethics approval by the Joint Ethics Committee of the Universities of Applied Sciences of Bavaria (No. GEHBa-202310-W-137).

### Supplementary Information

Supplementary Information.

## Data Availability

The dataset used during the current study in form of a list with code-numbers is available upon reasonable request (marcus.grohmann@hygiene-tk.de).
