# Supplementary material for: Impact on the German asymptomatic screening strategy based on actual user data from SARS-CoV-2 test centers
Source: Sci Rep. 2023 Nov 15;13:19959. doi: 10.1038/s41598-023-47262-x (PMC10652004; doi:10.1038/s41598-023-47262-x)
Supplement: Supplementary file 1 — Supplementary Information. [file 41598_2023_47262_MOESM1_ESM.pdf]

Supplement

Impact on the German asymptomatic screening strategy based on actual user data from SARS-CoV-2 test centers

Marcus Grohmann, Janina Grosch, Beate Conrady, Lena Schomakers and Anna Kristina Witte

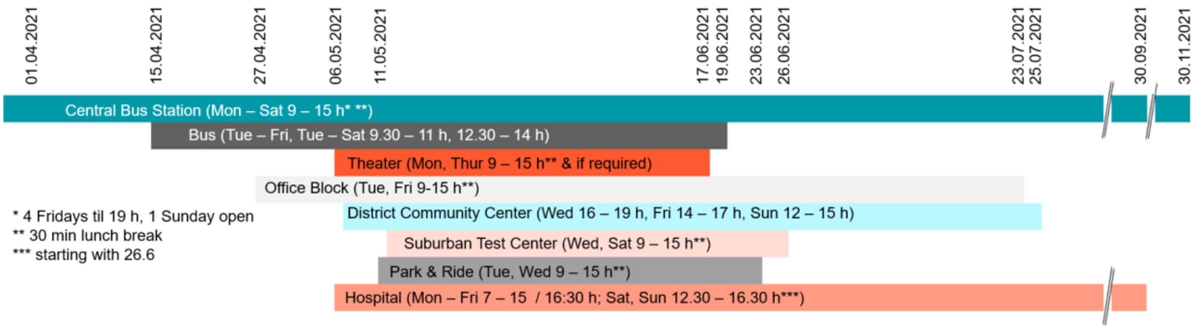

Supplemental Figure S1. Overview of opening periods and hours of all test centers.

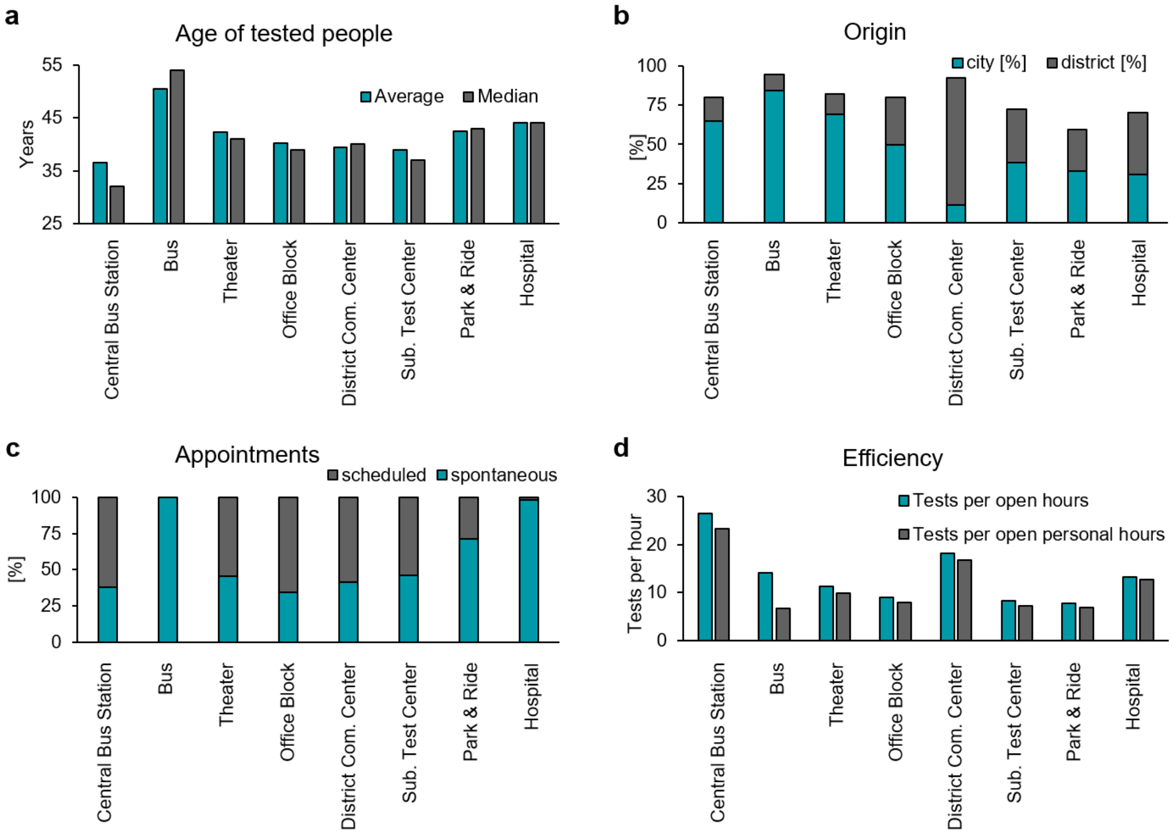

Supplemental Figure S2. Characteristics of people visiting test centers. a. Average (turquoise) and median (grey) of the visitors' ages: Visitors of the Central Bus station were significantly younger (37 years average; 32 years median), while visitors of the bus were significantly older (50 years average; 53 years median) compared to other locations. b. Proportion of visitors from the city Bamberg (turquoise)

and its rural district (grey): The mostly centrally located centers (Central Bus Station and Theater) and the Bus had the highest percentage of tests from visitors residing in the city while the Suburban Test Center, the test center at Park & Ride and at the hospital had less than 50 % of the tests from visitors from the city. The District Community Center located outside the city attracted mostly visitors residing in the urban district. c. Ratios of spontaneous (turquoise) and scheduled (grey) appointments for testing: The test center Park & Ride was used in a more spontaneous manner (71 % spontaneous) compared to the other locations with the exception of the test center in the Bus which did not offer appointments and the center at the local Hospital where the information to make appointments was not made publicly available. d. Efficiency of each test center indicated in performed tests per hours related to opening hours (turquoise) and hours in which personnel was employed/occupied: More tests were performed per open hour and per staff hour either in the larger test centers as the Central Bus Station and the Hospital or at the District Community Center, with fewer alternative options for testing nearby. In contrast, the Bus showed low staff efficiency per hour because the staff could not perform tests in the time taken travelling from one location to the next.

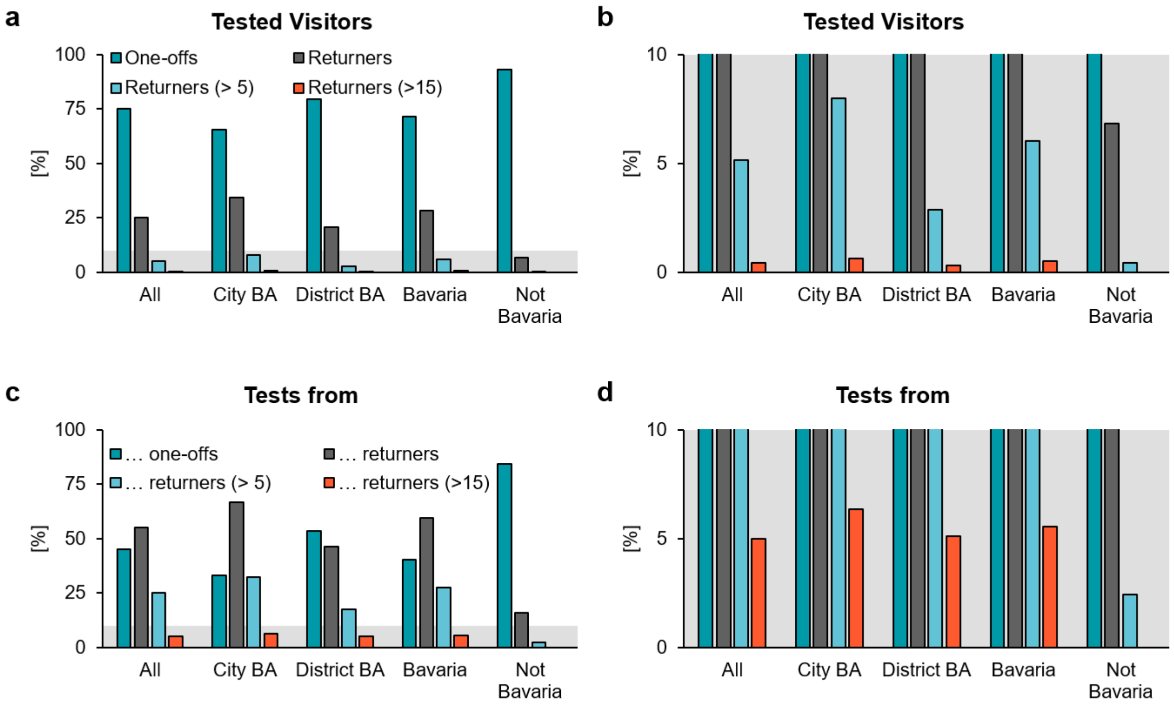

Supplemental Figure S3. Proportion of tested visitors (a, b) and tests (c, d) originating from one-offs and returners at the Central Bus Station. The highest proportion of returners and tests from returners can be traced to residents of the city Bamberg and no frequent returner (more than 15 times in the eight months study period) resided outside of Bavaria. The highest proportion of one-off visitors was individuals with no residence in Bavaria, whilst the lowest proportion of one-off visitors were Bamberg residents.

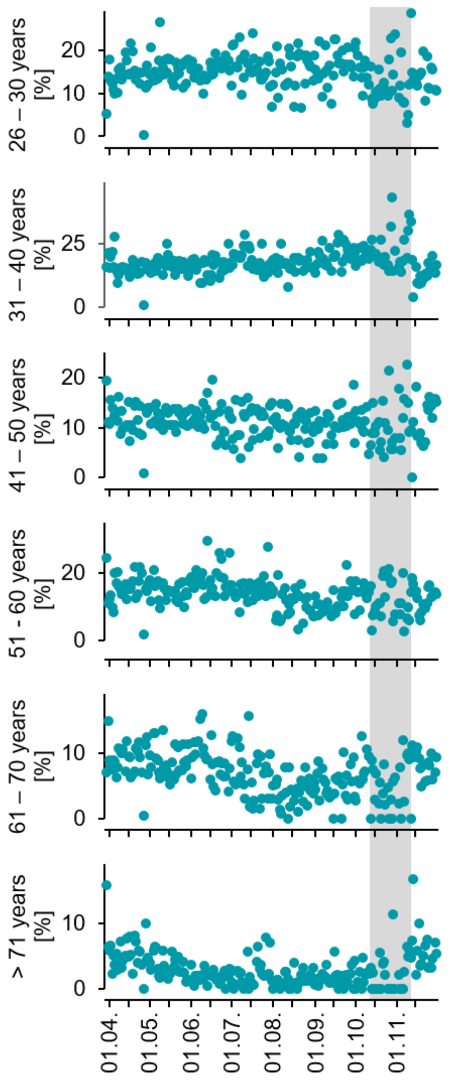

Supplemental Figure S4. Age groups of tested people over the study period. The grey area reflects the time where tests were fee-based for most individuals.

Supplemental Table S5. Summary of the estimated final negative binomial model parameters influencing the number of SARS-CoV-2 tests

Independent variables	**LR Chisq**	**Df**	**P value**	
Mitigation Measures	802.79	9	< 0.001	***
Average age	3.39	1	0.07	.
Weekday	68.95	6	< 0.001	***
Proportion of returners (>15 times)	1.32	1	0.25	

Significant. codes: 0 '***' 0.001 '**' 0.01 '*' 0.05 '.' 0.1 ' ' 1

Supplemental Table S6. The odds of individual measures compared to the intercept in terms of numbers of tests. The intercept includes the mitigation measure PT and the weekday Tuesday.

	**ODDS**	**Lower CI (2.5 %)**	**Upper CI (97.5 %)**	**P Value**
Intercept	51.37	32.50	81.21	< 0.001
Measures PTG	0.97	0.75	1.25	0.80
Measures G	2.92	2.31	3.69	< 0.001
Measures D	4.76	3.67	6.18	< 0.001
Measures DWTG	6.71	4.96	9.10	< 0.001
Measures DPTG	0.77	0.51	1.17	0.22
Measures DG	6.55	4.94	1.17	< 0.001
Measures DT	6.71	5.17	8.72	< 0.001
Measures DTG	6.58	5.15	8.43	< 0.001
Measures TG	3.54	2.80	4.47	< 0.001
Average age	0.99	0.98	1.00	0.06
Weekday Thursday	1.08	0.96	1.22	0.18
Weekday Friday	1.33	1.18	1.50	< 0.001
Weekday Wednesday	1.00	0.89	1.13	0.98
Weekday Monday	1.15	1.02	1.29	0.02
Weekday Saturday	1.47	1.30	1.66	< 0.001
Weekday Sunday	0.56	0.33	0.93	0.25
Proportion returner (>15)	0.99	0.98	1.01	0.25

N.B. Most of the mitigation measures were implemented as combinations:

P: Fee-based/paid testing period: SARS-CoV-2 PoC Antigen tests must be paid by most people (exceptions: people younger than 18, pregnant & breastfeeding women, people that cannot be vaccinated against SARS-CoV-2 due to medical reasons, SARS-CoV-2 infected people, which need testing for ending isolation, people participating on clinical studies for vaccinations against SARS-CoV-11/10/2021 – 12/11/2021

T: Tests required for many activities (changes between everyone must be tested, only neither vaccinated nor recovered must be tested, only tests for inside activities, only test for not vaccinated/recovered people necessary etc.): 17/04/2021 – 27/05/2021, 23/08/2021 – 10/10/2021 and 18/10/2021 – 30/11/2021

G: Gastronomy open: 10/05/2021 - 30/11/2021

D: *Katastrophenfall in Bayern*, disaster situation in Bavaria is determined by the Bavarian government between 29/03/2021 – 06/06/2021 and 10/11/2021 – 30/11/2021

W: '3G am Arbeitsplatz': People must be (1) vaccinated, (2) recovered or (3) negatively tested at the workplace; controls by employers (24/11/2021 – 30/11/2021)
